# Association between nutritional literacy and nutrition label use in Chinese community residents

**DOI:** 10.3389/fnut.2024.1380310

**Published:** 2024-06-27

**Authors:** Ling Yang, Yan Cui, Jing Du, Zihao Liu, Ying Duan, Qi Qi, Huaqing Liu, Min Zhang

**Affiliations:** ^1^School of Public Health, Bengbu Medical University, Bengbu, Anhui, China; ^2^School of Health Management, Bengbu Medical University, Bengbu, Anhui, China

**Keywords:** nutritional literacy, nutrition label, nutrition facts table, purported nutritional benefits, purported health benefits, China

## Abstract

**Introduction:**

Nutritional literacy (NL) has a critical influence on food choices. The objective of the present study was to examine the association of NL with nutrition label use.

**Methods:**

A cross-sectional study was conducted in Bengbu, China. In total, 955 adults were interviewed using a questionnaire designed for the present study to collect information on demographics, lifestyle, nutrition label use, and NL. Binary logistic regression models were used to calculate odds ratios (OR) and 95% confidence intervals (CI) for nutrition label use and its predictive variables.

**Results:**

In total, 40.4% of the participants reported looking at nutrition label when purchasing prepackaged foods. NL was significantly positively associated with nutrition label use and specifically with checking nutrition facts table, purported nutrition benefits and purported health benefits. In terms of specific facets of NL, nutrition knowledge, applying skills, and critical skills were associated with nutrition label use. After stratification by monthly income and education, the association between NL and nutrition label use was discovered only in individuals with low monthly income. Additionally, nutrition knowledge was associated with nutrition label use only in adults with high education level, whereas applying skills were associated with nutrition label use only in those with low education level.

**Conclusion:**

The use of nutrition label remains low among Chinese community residents, especially the purported nutritional benefits and purported health benefits. NL is positively associated with nutrition label use, especially with respect to functional and critical NL, with differences based on socioeconomic status. The findings highlight the need for NL interventions targeting individuals with different levels of education and income to encourage use of nutrition label in China.

## 1 Introduction

Nutrition label inform consumers of the nutritional information of prepackaged foods, enabling them to make informed decisions and encouraging healthy eating habits (1, 2). In China, the Chinese Food Nutrition Labeling Regulation, promulgated in 2008, stipulates that a nutrition label should inform consumers about a food’s nutritional content and characteristics and include a nutritional fact table (hereafter “nutritional facts”) and descriptions of the purported nutritional and health benefits (3).

Nutrition label use is associated with higher diet quality (4). Studies have shown that consumers who consult nutrition label when purchasing prepackaged foods report healthier nutrient consumption (5) and have higher dietary fiber intake (5); are more likely to choose fruits, vegetables and legumes (6); and consume less sodium (7) compared with individuals who do not use nutrition label. In addition, one study from Mexico indicated that nutrition label use was associated with chronic conditions and people with obesity, diabetes, and a combination of chronic conditions were less likely to use nutrition labels than people without these conditions (8). Therefore, nutrition labeling can be an essential tool for promoting healthy eating and preventing diet-related noncommunicable diseases such as obesity (9–11).

Several laws and regulations have been implemented in countries to promote nutrition label use. For example, the United States enacted the Nutrition Labeling and Education Act (12), and China issued the Chinese Food Nutrition Labeling Regulations as well as the General Principles of Nutrition Labeling of Prepackaged Foods (3), which recommend disseminating information on nutritious foods to consumers in order to enhance their healthy eating habits. However, the use of nutrition label in countries is not encouraging. For example, studies have shown that only 25.1% of individuals in Arab countries (13), only 59.3% of individuals in Israel (14), only 27.0% of individuals in the United Kingdom (15) and only 27.16% people in Nanjing use nutritional label when deciding what to buy (16). The use of nutritional label was influenced by several factors, particularly age, gender, place of residence, and education (13, 14). Females aged 30–49, married individuals were more likely to use nutrition labels (17, 18). Rural respondents were less aware of nutrition labeling and had lower usage and perceived benefits compared to urban respondents (19). In addition, this usage is affected by subjective factors. High perceptions of the importance of nutrition were significantly associated with frequency of using information on food labels (14). Some studies showed that consumers with a strong nutritional knowledge base are more likely to use nutritional labels (15, 16). However, there are inconsistent results (20–22). Hazali (21) found that nutrition knowledge was not associated with nutrition label use. Another study reported only an indirect effect of nutritional knowledge, indicating that attitude mediated the relationship between nutritional knowledge and label use (22). Hence, looking for key factors affecting the nutrition label use is important for making out specific and targeted intervention.

Nutritional literacy (NL), rooted in the discipline of nutritional science and health literacy, is defined as the ability to access, understand, and apply nutrition-related information to make sound nutritional decisions (20, 21). In the most recent decade, NL attracted researchers’ increasing attention in the field of health promotion, and a series of measure tools for NL have been developed (23, 24). Therefore, NL and dietary behavior and related health issues has begun to widely researched. Some studies reported that NL is strongly associated with eating behaviors and influences dietary choices and health (25–28). Therefore, NL, which is regarded as asset from a public health perspective, might be a key factor mediating nutrition label use. However, to date, their relationship is still not well documented.

In China, our previous study was the first to develop a nutritional literacy measurement scale for Chinese adults, with a good reliability and validity (29). This study was to explore the association between NL and nutrition label use in residents of a Chinese community, and the results can provide new evidence on making interventions or measurements targeted specific nutrition literacy to promote the use of nutrition label for adults.

## 2 Materials and methods

### 2.1 Participants and procedure

This study was a cross-sectional survey conducted in August 2022 in Yuhui District, Bengbu, China, to examine the relationship between NL and nutrition label use among community-dwelling people aged 18 years and older. Participants were recruited from all 32 communities in the district, and about 30 individuals were randomly interviewed each community. According to the Seventh National Population Census of Bengbu City, the resident population in Yuhui District accounts for 9.88% of the population in Bengbu. The inclusion criteria for participants in the study were being older than 18 years, having the ability to travel and shop independently, and living in their community for at least the 6 months prior to the survey. All participants participated voluntarily and provided written informed consent. Approval for this study was obtained from the Ethics Committee of Bengbu Medical University.

A structured questionnaire was designed by the study authors to gather data on the participants’ demographic information, lifestyle, nutrition label use, and NL. One-to-one and face-to-face interviews were administered by investigators trained in uniform norms. A total of 992 participants were interviewed and 992 questionnaires were collected, with a response rate of 100%. Thirty-seven questionnaires were invalid and excluded due to missing data, the remaining 955 were included in the final study, with a validity rate of 96.27%.

### 2.2 NL assessment

The participants’ NL was assessed using the short-form nutrition literacy measurement tool (30) developed by our team, which consists of items in three levels (functional NL[The ability to understand, obtain, and apply nutritional information or nutrition services], interactive NL[the ability to interact with food-related environments and avoid unhealthy dietary behaviors] and critical NL[the ability to critically reflect on nutritional knowledge or dietary advice in relation to individual needs]). The functional NL includes nutrition knowledge, understanding, obtaining skills and applying skills (29). The tool has satisfactory validity in Chinese adults (29, 30). The short-form nutrition literacy measurement tool contains 12 items, each scored on a five-point Likert scale. The scores for each item are summed to yield a total NL score, with higher scores indicating higher NL. The Cronbach’s coefficient is 0.892 in this study.

### 2.3 Nutrition label use

Nutrition label use was determined by responses to the following three questions on nutrition facts, purported nutrition benefits, and purported health benefits: “Do you always check the nutrition facts when purchasing prepackaged foods?” “Do you always check the nutrition claims when purchasing prepackaged foods?” and “Do you always check the claimed nutritional benefits when purchasing prepackaged foods?” For each question, two responses were possible: “Yes” and “No.” The responses for these three items were summed; nutrition label use was thus assessed on a 0–3 scale. An individual was identified as using nutrition label if they scored higher than 1.

### 2.4 Covariates

To control for the effects of potential confounding factors, we adjusted for the following covariates: gender (male or female), age (18–44 years, 45–64 years, or ≥ 65 years), body mass index (BMI), education (less than a high school diploma, high school diploma, or college degree or higher); retirement status (yes or no); monthly income (<2,000 RMB, 2,000–4,000 RMB, or ≥ 4,000 RMB), smoking habit (yes or no), alcohol consumption (yes or no), and daily exercise duration (<1 h, 1–2 h, or ≥ 3 h).

### 2.5 Statistical analysis

As a continuous variable, NL is presented as means ± standard deviations, and a *t*-test was used to evaluate differences in NL between “yes” and “no” for nutrition label use, nutrition facts table use, purported nutritional benefits, and purported health benefits use. As Categorical variables, gender, age, BMI, education, retirement status, monthly income, smoking habit, alcohol consumption, and daily exercise duration were presented as frequencies and percentages in this study, and chi-square tests were used to evaluate differences across groups. After adjusting for confounders (i.e., gender, age, BMI, education, retirement status, monthly income, smoking habit, alcohol consumption, and daily exercise duration), binary logistic regression was performed to calculate the odds ratio (OR) and 95% confidence interval (CI) for the association between NL and nutrition label use. We also conducted subgroup analyses based on educational attainment and monthly income to analyze the relationship between NL and nutrition label use among different groups.

Confirmatory factor analysis (CFA) was used to assess the construct validity of the short-form nutrition literacy measurement tool in the community residents and to confirm the fit of the model. The root mean square error approximation (RMSEA) value of less than 0.08 suggesting a high goodness of fit. The model fit index of more than 0.9 indicates an acceptable fit, and the model fit indices mainly include the normal fit index (NFI), the comparative fit index (CFI), the incremental fit index (IFI), the goodness-of-fit index (GFI), the Tucker-Lewis index (TLI), and the adjusted goodness-of-fit index (AGFI) (31). In addition, an average variance extracted (AVE) of >0.5 and composite reliability (CR) of >0.6 were used to assessed the item-scale convergent and discriminant validity.

Data were analyzed using AMOS 24.0 and SPSS 26.0, and *p* < 0.05 was considered statistically significant.

## 3 Results

### 3.1 Participant characteristics

Table 1 shows the demographic characteristics of the 955 participants. The participants had a mean age of 61.34 ± 16.475 years; 156 (16.3%) were 18–44 years, 315 (33.0%) were 45–64 years, and 484 (50.7%) were ≥ 65 years. Overall, 916 (46.6%) were men, whereas 395 (41.4%) had a BMI of 18.5–23.9 kg/m^2^. A total of 566 (59.3%) had less than a high school diploma, and 525 (55.0%) had a monthly income of 2000–4,000 RMB.

**Table 1:** Nutrition label use in participant subgroups.

Variables	N (%)	Total nutrition label use n (%)	χ^2^	Nutrition facts table use n (%)	χ^2^	Purported nutritional benefits use n (%)	χ^2^	Purported health benefits use n (%)	χ^2^
Total	955 (100)	386 (40.4)		361 (37.8)		121 (12.7)		82 (8.6)	
Age group (years)			25.74***		29.36***		0.54		3.98
18–44	156 (16.3)	88 (56.4)		87 (55.8)		22 (14.1)		17 (10.9)	
45–64	315 (33.0)	134 (42.5)		121 (38.4)		37 (11.7)		32 (10.2)	
65–95	484 (50.7)	164 (33.9)		153 (31.6)		62 (12.8)		33 (6.8)	
Gender			0.01		0.03		6.06*		0.17
Male	445 (46.6)	179 (40.2)		167 (37.5)		69 (15.5)		40 (9.0)	
Female	510 (53.4)	207 (40.6)		194 (38.0)		52 (10.2)		42 (8.2)	
BMI (kg/m^2^)			8.95*		7.03		5.04		3.57
<18.5	30 (3.1)	12 (40.0)		12 (40.0)		7 (23.3)		3 (10.0)	
18.5–23.9	395 (41.4)	168 (42.7)		158 (40.0)		47 (11.9)		41 (10.4)	
24–27.9	375 (39.3)	160 (42.7)		147 (39.2)		52 (13.9)		29 (7.7)	
≥28	155 (16.2)	46 (29.7)		44 (28.4)		15 (9.7)		9 (5.8)	
Education			87.87***		90.88***		21.30***		12.85**
Less than a high school diploma	566 (59.3)	159 (28.1)		144 (25.4)		50 (8.8)		37 (6.5)	
High School diploma	211 (22.1)	121 (57.3)		114 (54.0)		44 (20.9)		18 (8.5)	
College degree or higher	178 (18.6)	106 (59.6)		103 (57.9)		27 (15.2)		27 (15.2)	
Retirement			0.05		0.63		9.30**		0.11
Yes	484 (50.7)	194 (40.1)		177 (36.6)		77 (15.9)		43 (8.9)	
No	471 (49.3)	192 (40.8)		184 (39.1)		44 (9.3)		39 (8.3)	
Monthly income			37.31***		30.60***		26.34***		14.49**
<2000 RMB	314 (32.9)	88 (28.0)		84 (26.8)		15 (4.8)		12 (3.8)	
2000–4,000 RMB	525 (55.0)	231 (44.0)		215 (41.0)		87 (16.6)		60 (11.4)	
≥4,000 RMB	116 (12.1)	67 (57.8)		62 (53.4)		19 (16.4)		10 (18.6)	
Smoking			0.38		0.47		0.57		0.02
No	740 (77.5)	303 (40.9)		284 (38.4)		97 (13.1)		63 (8.5)	
Yes	215 (22.5)	132 (61.4)		77 (35.8)		24 (11.2)		19 (8.8)	
Drinking			1.48		1.62		0.10		0.48
No	715 (74.9)	281 (39.3)		262 (36.6)		92 (12.9)		64 (9.0)	
Yes	240 (25.1)	105 (43.8)		99 (41.3)		29 (12.1)		18 (7.5)	
Exercise duration/d (h)			3.85		2.86		23.53***		2.15
<1	144 (15.1)	59 (41.0)		55 (38.2)		16 (11.1)		11 (7.6)	
1-	322 (33.7)	124 (38.5)		120 (37.3)		20 (6.2)		25 (7.8)	
2-	271 (28.4)	122 (45.0)		112 (41.3)		43 (15.9)		22 (8.1)	
≥3	218 (22.8)	81 (37.2)		74 (33.9)		42 (19.3)		24 (11.0)	

*P < 0.05, **p < 0.01, ***p < 0.001.

As detailed in Tables 1, 386 (40.4%) participants reported using nutrition labeling when purchasing prepackaged foods. Breaking this down by information type revealed that 361 (37.8%) used nutrition facts, 121 (12.7%) used purported nutritional benefits, and 82 (8.6%) used purported health benefits.

Age of 18–44 years, BMI of 18.5–27.9 kg/m^2^, education level of college or higher, and monthly income ≥4,000 RMB were associated with more frequent use of nutrition label on prepackaged foods.

### 3.2 Confirmatory factor analysis of short-form nutrition literacy measurement tool

CFA was preformed to evaluate the structural validity of the short-form nutrition literacy measurement tool. Table 2 shows good model–data fit and convergent validity. The RMSEA value of the tool was 0.061, and the GFI, AGFI, CFI, IFI, TLI, and NFI values ranged from 0.938 to 0.977. The convergent validity results were shown in Table 3 presents. In most dimensions, the AVE and CR values were above 0.5 and 0.6, respectively, with the exception of the dimension of applying skills, where the AVE value was 0.392 and CR value was 0.531.

**Table 2:** Construct validity for the goodness-of-fit indices of the short-form nutrition literacy measurement tool.

χ^2^/df	RMSEA	GFI	AGFI	CFI	IFI	TLI	NFI
4.58	0.061	0.969	0.938	0.977	0.977	0.962	0.971

**Table 3:** AVE and CR values for the six dimensions of the short-form nutrition literacy measurement tool.

	AVE	CR (Spearman-Brown coefficient)
Knowledge	0.519	0.679
Understanding	0.810	0.895
Obtaining skills	0.662	0.796
Applying skills	0.392	0.531
Interactive skills	0.742	0.852
Critical skills	0.707	0.828

### 3.3 Nutrition label use given NL

Table 4 shows that the mean NL score was 37.20 ± 9.55, with the mean score being 24.87 ± 6.37 in functional NL (knowledge [8.07 ± 1.43], understanding [5.61 ± 2.27], obtaining skills [5.63 ± 2.25] and applying skills [5.56 ± 2.09]), interactive NL [6.23 ± 2.28] and critical NL [6.10 ± 2.17]. Additionally, those who used nutrition label had a higher mean NL score and higher scores for the functional NL, interactive NL, and critical NL (*p* < 0.05).

**Table 4:** Nutritional literacy scores of Chinese adults segmented in accordance with type of nutrition label used.

Variables	Mean ± SD	Total nutrition label use			Nutrition facts table use			Purported nutritional benefits use			Purported health benefits use		
		None (*n* = 569)	Yes (*n* = 386)	*t*	None (*n* = 594)	Yes (*n* = 361)	*t*	None (*n* = 834)	Yes (*n* = 121)	*t*	None (*n* = 873)	Yes (*n* = 82)	*t*
Nutrition literacy	37.20 ± 9.55	34.09 ± 8.61	41.79 ± 9.00	−13.310***	34.34 ± 8.68	41.92 ± 9.02	−12.900***	36.23 ± 9.32	43.93 ± 8.32	−8.611***	36.72 ± 9.42	42.32 ± 9.47	−5.14***
Functional NL	24.87 ± 6.37	22.98 ± 5.69	27.67 ± 6.28	−11.997***	23.13 ± 5.75	27.74 ± 6.30	−11.328***	24.25 ± 6.23	29.14 ± 5.61	−8.159***	24.55 ± 6.30	28.33 ± 6.00	−5.213***
Knowledge	8.07 ± 1.43	7.78 ± 1.42	8.49 ± 1.33	−7.777***	7.8 ± 1.43	8.52 ± 1.31	−7.812***	7.98 ± 1.45	8.65 ± 1.09	−4.872***	8.04 ± 1.43	8.37 ± 1.37	−1.972*
Understanding	5.61 ± 2.27	5.16 ± 2.14	6.27 ± 2.29	−7.641***	5.21 ± 2.16	6.27 ± 2.30	−7.203***	5.44 ± 2.24	6.82 ± 2.1	−6.395***	5.51 ± 2.25	6.68 ± 2.17	−4.522***
Obtaining skills	5.63 ± 2.25	5.13 ± 2.14	6.37 ± 2.19	−8.745***	5.18 ± 2.15	6.38 ± 2.21	−8.319***	5.46 ± 2.23	6.82 ± 2.02	−6.839***	5.53 ± 2.24	6.7 ± 2.08	−4.537***
Applying skills	5.56 ± 2.09	4.91 ± 1.86	6.53 ± 2.02	−12.608***	4.95 ± 1.89	6.57 ± 2.00	−12.369***	5.38 ± 2.04	6.85 ± 1.92	−7.472***	5.47 ± 2.08	6.59 ± 1.91	−4.69***
Interactive NL	6.23 ± 2.28	5.64 ± 2.24	7.09 ± 2.05	−10.308***	5.68 ± 2.24	7.12 ± 2.05	−10.157***	6.06 ± 2.29	7.36 ± 1.86	−6.984***	6.14 ± 2.27	7.10 ± 2.15	−3.647***
Critical NL	6.10 ± 2.17	5.47 ± 2.09	7.03 ± 1.95	−11.774***	5.52 ± 2.10	7.06 ± 1.94	−11.535***	5.91 ± 2.13	7.43 ± 2.00	−7.396***	6.03 ± 2.15	6.89 ± 2.22	−3.454*

*P < 0.05, **P < 0.01, ***P < 0.001. Nutrition literacy includes two domains: nutrition cognition (knowledge and understanding) and nutrition skills (obtaining skills, applying skills, interactive skills, and critical skills).

### 3.4 Association of NL with nutrition label use when purchasing packaged foods

As shown in Table 5, adjusting for age, gender, BMI, education, retirement status, monthly income, smoking habits, alcohol consumption, and daily exercise duration, we discovered that NL was strongly positively associated with nutrition label use (OR = 1.09, 95% CI = 1.07–1.11); this relationship was strongest with respect to functional NL (OR = 1.06, 95%CI = 1.03–1.10) [specifically nutrition knowledge (OR = 1.19, 95% CI = 1.06–1.34) and applying skills (OR = 1.30, 95% CI = 1.18–1.41)], and critical NL (OR = 1.21, 95% CI =1.10–1.34). These skills were associated with nutrition fact use. NL was also associated with purported nutritional benefit use (OR = 1.10, 95% CI = 1.07–1.13) and purported health benefit use (OR = 1.06, 95% CI = 1.03–1.09); however, these associations were observed only in the applying skills dimension.

**Table 5:** Binary logistic regression of the association between NL and nutrition label use when purchasing packaged foods.

Variables	Total nutrition label use	Nutrition facts table use	Purported nutritional benefits use	Purported health benefits use
	OR (95% CI)	OR (95% CI)	OR (95% CI)	OR (95% CI)
Nutritional literacy	1.09 (1.07–1.11) ***	1.09 (1.07–1.11) ***	1.10 (1.07–1.13) ***	1.06 (1.03–1.09) ***
Functional NL	1.06 (1.03–1.10) ***	1.05 (1.02–1.09) ***	1.10 (1.05–1.15) ***	1.08 (1.02–1.13) **
Knowledge	1.19 (1.06–1.34) **	1.21 (1.07–1.36) **	1.16 (0.96–1.39)	0.98 (0.82–1.17)
Understanding	0.85 (0.72–1.01)	0.84 (0.71–0.99) *	1.07 (0.85–1.36)	1.05 (0.80–1.38)
Obtaining skills	1.10 (0.93–1.31)	1.09 (0.91–1.30)	1.05 (0.82–1.34)	1.09 (0.82–1.44)
Applying skills	1.30 (1.18–1.41) ***	1.27 (1.16–1.40) ***	1.18 (1.04–1.34) *	1.17 (1.01–1.35) *
Interactive NL	1.08 (0.99–1.19)	1.09 (0.99–1.20)	1.07 (0.92–1.23)	1.04 (0.89–1.21)
Critical NL	1.21 (1.10–1.34) ***	1.21 (1.10–1.34) ***	1.16 (1.00–1.34) *	1.00 (0.85–1.18)

*P < 0.05, **P < 0.01, ***P < 0.001. OR, odds ratio; 95% CI, 95% confidence interval. The OR (95% CI) was calculated using binary logistic regression when adjusting for age, gender, BMI, education, retirement status (yes or no), monthly income (RMB), smoking (yes or no), drinking (yes or no) and daily exercise duration.

### 3.5 Subgroup analysis

As detailed in Tables 6, a positive correlation was found between NL and nutrition label use in individuals with a monthly income of <2,000 RMB (OR = 1.12, 95% CI = 1.08–1.16) or 2,000–4,000 RMB (OR = 1.09, 95% CI = 1.06–1.11) but not in individuals with a monthly income of ≥4,000 RMB. The association applied to nutrition fact table use, purported nutritional benefit use, and purported health benefit use. More specifically, nutrition knowledge was associated with nutrition label use in individuals with a college education or greater (OR = 1.29, 95% CI = 1.01–1.66) and those with a monthly income of <2,000 RMB (OR = 1.38, 95% CI = 1.07–1.76) but not in individuals with a lower educational level or higher monthly income. The association of applying skills with nutrition label use existed only in those with less than a high school diploma (OR = 1.37, 95% CI = 1.21–1.56) and those with a monthly income of <2,000 RMB (OR = 1.42, 95% CI = 1.17–1.72) or 2,000–4,000 RMB (OR = 1.27, 95% CI = 1.13–1.43). However, for adults only with a high school diploma (OR = 1.23, 95% CI = 1.01–1.50) and with a monthly income of ≥4,000 RMB (OR = 1.43, 95% CI = 1.06–1.93), interactive NL were significantly positively associated with nutrition label use.

**Table 6:** Subgroup analysis of associations between NL and nutrition label use when purchasing prepackaged foods.

Subgroups	Variables	Total nutrition label use	Nutrition facts table use	Purported nutritional benefits use	Purported health benefits use
		OR (95% CI)	OR (95% CI)	OR (95% CI)	OR (95% CI)
Education					
Less than a high school diploma	Nutritional literacy	1.09 (1.07–1.12) ***	1.10 (1.07–1.13) ***	1.14 (1.10–1.19) ***	1.04 (1.00–1.08)
	Functional NL	1.09 (1.04–1.14) ***	1.09 (1.04–1.14) ***	1.15 (1.06–1.23) ***	1.12 (1.04–1.21) **
	Knowledge	1.15 (0.97–1.37)	1.18 (0.98–1.41)	1.21 (0.91–1.62)	0.98 (0.73–1.30)
	Understanding	0.85 (0.68–1.08)	0.87 (0.69–1.11)	1.08 (0.75–1.54)	1.19 (0.79–1.78)
	Obtaining skills	1.16 (0.91–1.47)	1.11 (0.87–1.42)	1.10 (0.76–1.59)	1.00 (0.66–1.50)
	Applying skills	1.37 (1.21–1.56) ***	1.39 (1.22–1.59) ***	1.27 (1.05–1.53) *	1.31 (1.06–1.62) *
	Interactive NL	0.98 (0.87–1.11)	1.00 (0.88–1.13)	1.03 (0.84–1.26)	0.90 (0.73–1.12)
	Critical NL	1.24 (1.09–1.42) **	1.24 (1.08–1.43) **	1.26 (1.00–1.58) *	0.94 (0.75–1.19)
High school diploma	Nutritional literacy	1.06 (1.02–1.11) **	1.05 (1.01–1.10) *	1.10 (1.03–1.17) **	1.11 (1.02–1.20) *
	Functional NL	1.00 (0.94–1.08)	0.98 (0.92–1.05)	1.14 (1.04–1.26) **	1.09 (0.96–1.24)
	Knowledge	1.15 (0.90–1.47)	1.16 (0.91–1.47)	1.03 (0.71–1.50)	0.77 (0.45–1.34)
	Understanding	0.85 (0.60–1.18)	0.79 (0.57–1.10)	1.05 (0.69–1.60)	0.99 (0.51–1.91)
	Obtaining skills	1.04 (0.72–1.48)	1.07 (0.75–1.51)	1.25 (0.79–1.95)	1.09 (0.55–2.20)
	Applying skills	1.17 (0.97–1.42)	1.13 (0.94–1.36)	1.21 (0.95–1.55)	1.43 (1.00–2.06)
	Interactive NL	1.23 (1.01–1.50) *	1.22 (1.00–1.48) *	1.11 (0.85–1.44)	1.06 (0.72–1.57)
	Critical NL	1.13 (0.93–1.38)	1.13 (0.96–1.41)	0.92 (0.72–1.19)	1.24 (0.85–1.81)
College degree or higher	Nutritional literacy	1.13 (1.08–1.19) ***	1.11 (1.06–1.17) ***	1.06 (1.01–1.12) *	1.06 (1.01–1.12) *
	Functional NL	1.08 (0.99–1.17)	1.07 (0.98–1.16)	0.95 (0.84–1.08)	1.00 (0.89–1.12)
	Knowledge	1.29 (1.01–1.66) *	1.29 (1.01–1.65) *	1.38 (0.81–2.35)	1.07 (0.77–1.50)
	Understanding	0.94 (0.60–1.47)	0.87 (0.56–1.34)	1.06 (0.56–2.01)	0.99 (0.54–1.80)
	Obtaining skills	1.02 (0.63–1.64)	1.08 (0.68–1.71)	0.83 (0.42–1.67)	1.23 (0.63–2.40)
	Applying skills	1.17 (0.91–1.50)	1.16 (0.92–1.47)	0.81 (0.58–1.14)	0.78 (0.56–1.09)
	Interactive NL	1.18 (0.91–1.53)	1.15 (0.89–1.48)	1.28 (0.82–2.02)	1.42 (0.94–2.13)
	Critical NL	1.33 (1.00–1.77) *	127 (0.97–1.67)	1.33 (0.86–2.00)	0.99 (0.70–1.41)
Monthly income					
<2,000 RMB	Nutritional literacy	1.12 (1.08–1.16) ***	1.12 (1.07–1.16) ***	1.21 (1.09–1.34) ***	1.10 (1.01–1.20) *
	Functional NL	1.11 (1.04–1.18) **	1.10 (1.03–1.18) **	1.09 (0.91–1.29)	1.19 (1.03–1.38) *
	Knowledge	1.38 (1.07–1.76) *	1.36 (1.06–1.75) *	1.67 (0.64–4.33)	1.08 (0.64–1.84)
	Understanding	0.73 (0.52–1.03)	0.72 (0.51–1.01)	0.70 (0.29–1.65)	1.77 (0.83–3.80)
	Obtaining skills	1.31 (0.90–1.90)	1.30 (0.89–1.89)	0.99 (0.38–2.57)	0.85 (0.38–1.89)
	Applying skills	1.42 (1.17–1.72) ***	1.46 (1.20–1.77) ***	1.64 (1.01–2.69) *	1.21 (0.76–1.91)
	Interactive NL	1.02 (0.85–1.22)	1.04 (0.86–1.24)	0.69 (0.42–1.15)	1.03 (0.68–1.54)
	Critical NL	1.27 (1.03–1.57) *	1.26 (1.01–1.56) *	4.24 (1.93–16.30) ***	0.91 (0.55–1.51)
2,000–4,000 RMB					
	Nutritional literacy	1.09 (1.06–1.11) ***	1.08 (1.05–1.11) ***	1.11 (1.07–1.15) ***	1.05 (1.02–1.09) *
	Functional NL	1.05 (1.01–1.10) *	1.04 (1.00–1.09)	1.12 (1.06–1.19) ***	1.06 (1.00–1.13)
	Knowledge	1.09 (0.94–1.27)	1.12 (0.96–1.30)	1.03 (0.83–1.28)	0.93 (0.75–1.14)
	Understanding	0.96 (0.77–1.19)	0.98 (0.79–1.22)	1.15 (0.86–1.54)	1.02 (0.74–1.41)
	Obtaining skills	1.01 (0.80–1.27)	0.96 (0.77–1.21)	1.08 (0.80–1.45)	1.10 (0.79–1.53)
	Applying skills	1.27 (1.13–1.43) ***	1.23 (1.09–1.38) **	1.18 (1.01–1.37) *	1.19 (1.00–1.41)
	Interactive NL	1.08 (0.95–1.22)	1.08 (0.96–1.23)	1.11 (0.93–1.32)	1.00 (0.83–1.21)
	Critical NL	1.22 (1.07–1.39) **	1.23 (1.08–1.40) **	1.08 (0.91–1.28)	1.06 (0.87–1.28)
≥4,000 RMB					
	Nutritional literacy	1.06 (1.00–1.12)	1.05 (1.00–1.11)	1.04 (0.97–1.13)	1.06 (0.96–1.17)
	Functional NL	0.98 (0.88–1.08)	0.99 (0.90–1.09)	1.02 (0.89–1.19)	0.97 (0.79–1.20)
	Knowledge	1.39 (0.91–2.11)	1.41 (0.93–2.13)	2.54 (1.06–6.08) *	2.28 (0.72–7.16)
	Understanding	0.74 (0.43–1.29)	0.59 (0.34–1.04)	1.55 (0.66–3.61)	2.39 (0.64–8.93)
	Obtaining skills	0.91 (0.53–1.55)	1.15 (0.67–1.96)	0.43 (0.16–1.19)	0.31 (0.06–1.62)
	Applying skills	1.17 (0.86–1.60)	1.21 (0.90–1.65)	1.08 (0.68–1.73)	0.68 (0.37–1.26)
	Interactive NL	1.43 (1.06–1.93) *	1.35 (1.01–1.80)*	1.36 (0.88–2.08)	2.22 (01.06–4.63) *
	Critical NL	0.99 (0.74–1.33)	1.00 (0.75–1.34)	0.85 (0.57–1.26)	0.72 (0.39–1.34)

*P < 0.05, **P < 0.01, ***P < 0.001. OR, odds ratio; 95% CI, 95% confidence interval. The OR (95% CI) was calculated using binary logistic regression when adjusting for age, gender, BMI, education, retirement status (yes or no), monthly income (RMB), smoking (yes or no), drinking (yes or no) and daily exercise duration.

## 4 Discussion

The present study is among the first to explore the associations between NL and nutrition label use in Chinese community residents when they are purchasing prepackaged foods. The results of this analysis reveal that NL was positively associated with nutrition label use. However, only 1 in 10 of the surveyed individuals checked purported nutrition or health benefits, despite the fact that nearly two in five adults checked nutrition label. This indicates that usage of nutrition label by Chinese adults remains low, suggesting an area of focus for health education reforms. Improving NL can help to promote the utilization of nutrition label and encourage healthy food choices by adults.

The results of this study are consistent with the results of another study, which reported that more than 60% of urban residents from 10 provinces in China rarely or never used nutrition label (19). Moreover, nutrition facts, rather than purported nutritional or health benefits, were found to be the most frequently used item on nutrition label. As in other countries, nutrition facts in China show calories and daily percentages of key nutrients per serving in a tabular format (32). Consumers attempting to adjust their dietary intake are more inclined to read nutrition facts table when purchasing prepackaged foods than are other consumers (33). Purported nutritional and health benefits are a permitted form of scientific promotion. However, many people consider nutrition benefits and purported health benefits for food ingredients as a form of promotion or exaggeration rather than a presentation of actual nutritional value (34). The Chinese public’s distrust of nutrition label points to a critical unmet NL need.

The results of the present study’s analysis revealed that NL (specifically functional NL and critical NL) is significantly positively associated with nutrition label use. NL is a measure of the ability to make sound nutritional decisions and is associated with a healthy dietary behavior. Additionally, nutrition knowledge is strongly associated with nutrition label use (32). Moreover, nutrition knowledge is positively associated with perceptions of the significance on nutrition label use (35). Increasing the perceptions of the importance of nutrition label may promote their use (14), and education related to nutrition knowledge in China must thus be improved. However, knowledge is frequently not enough to change behavior (36). One study reported that students’ dietary practices were unchanged by increases in their nutritional knowledge alone (37). Additionally, food preparation skills can positively influence eating behaviors (26). There is a great necessity from moving nutritional knowledge into skills related to behavior changes (38). Finally, the findings of the present study suggest that improving functional NL (especially nutritional knowledge and applying skills) and critical NL may effectively increase nutrition label use.

Consistent with the findings of a previous study (39), the present study discovered that nutrition label use differed in individuals with differing education and income levels. To further explore the relationship between NL and nutrition label use, we stratified participants by education and monthly income. The analysis revealed a positive association between NL and nutrition label use in adults with low or moderate income. These results are consistent with the findings of Speirs et al., who reported that in adults with low incomes, higher health literacy is associated with more frequent food label use (40). Individuals with lower incomes generally have lower NL (41) and lower rates of nutrition label use (18), trends also observed in the present study. Our findings underscore the necessity of targeting nutrition interventions at individuals with lower incomes.

In general, individuals with higher levels of education have greater nutrition knowledge, have superior understanding of nutritional information (42, 43), have higher nutrition label literacy (44), and are more likely to use nutrition label (39). However, in the present study, applying skills were associated with nutrition label use only in the group with a low level of education, although the association between nutrition knowledge and nutrition label use was found only in the high-education-level group. These findings may be due to highly educated people in China not having enough free time to apply their nutritional knowledge to healthy eating. For comparison, DEDIPAC studies on sedentary behaviors in Western countries revealed that individuals with the lowest level of education prepared food themselves for significantly more hours per day than did individuals with the highest level of education (45). Thus, lack of time may pose a major barrier to the use of food label information (35). The present study corroborates the findings of earlier studies and highlights the necessity of targeting nutrition interventions at those with higher levels of education.

The present study is among the first to examine the association of NL with nutrition label use. However, our study has some limitations. First, the study’s cross-sectional design does not permit causal inferences to be made. Second, because self-reported data were used in this study, discrepancies may have existed between participants’ self-reports and actual practice, potentially biasing the results. Moreover, this study identified the utilization of nutritional labels by subjective questions but not skill and practical questions. Finally, although the short-form nutrition literacy measurement tool showed good model–data fit and convergent validity, however, the convergent validity for applying skills was low and future research needs to improve convergent validity in terms of applying skills.

## 5 Conclusion

The present study identified that less than one-half of Chinese adults use the nutrition label when they purchase prepackaged foods. And they mainly looking at nutrition facts table, and less frequently looking at purported nutritional benefits and purported health benefits. Additionally, those who used nutrition label had a higher mean NL scores. NL, especially functional NL and critical NL, is positively associated with nutrition label use, with differences based on socioeconomic status. This association exist only in individuals with low monthly income. However, applying skills are associated with nutrition label use only in those with low education level. These findings highlight the importance for nutritional literacy interventions that are targeted to individuals with specific levels of education and income to promote nutrition label use in the Chinese population.

## Data availability statement

The raw data supporting the conclusions of this article will be made available by the authors, without undue reservation.

## Ethics statement

All participants participated voluntarily and provided written informed consent.

## Author contributions

LY: Conceptualization, Data curation, Formal analysis, Writing – original draft. YC: Software, Validation, Visualization, Writing – review & editing. JD: Conceptualization, Methodology, Project administration, Writing – review & editing. ZL: Data curation, Investigation, Writing – review & editing. YD: Data curation, Validation, Writing – review & editing. QQ: Investigation, Methodology, Validation, Writing – review & editing. HL: Conceptualization, Funding acquisition, Methodology, Resources, Writing – review & editing. MZ: Conceptualization, Funding acquisition, Writing – review & editing.
